# Gastric cancer-derived exosomal let-7 g-5p mediated by *SERPINE1* promotes macrophage M2 polarization and gastric cancer progression

**DOI:** 10.1186/s13046-024-03269-4

**Published:** 2025-01-02

**Authors:** Zhenzhen Ye, Jianfeng Yi, Xiangyan Jiang, Wengui Shi, Hao Xu, Hongtai Cao, Long Qin, Lixin Liu, Tianming Wang, Zhijian Ma, Zuoyi Jiao

**Affiliations:** 1 Department of General Surgery, The Second Clinical Medical School, The Second Hospital of Lanzhou University, Lanzhou University, Lanzhou, Gansu 730000 China; 2 The First School of Clinical Medicine, Gansu University of Chinese Medicine, Lanzhou, Gansu 730000 China; 3 Research Center of Traditional Chinese Medicine, Lanzhou, Gansu 730000 China; 4 The First School of Clinical Medicine, Lanzhou University, Lanzhou, Gansu 730000 China; 5 Cuiying Biomedical Research Center, Lanzhou University Second Hospital, Lanzhou, Gansu China; 6 Department of Hepatobiliary Surgery, The First Affiliated Hospital of Zhejiang Chinese Medical University, Zhejiang Provincial Hospital of Chinese Medicine, Hangzhou, Zhejiang 310006 China

**Keywords:** Gastric cancer, *SERPINE1*/PAI-1, let-7 g-5p, Cancer-derived exosome, M2 polarization

## Abstract

**Background:**

Tumor-associated macrophages (TAMs), particularly M2-polarized TAMs, are significant contributors to tumor progression, immune evasion, and therapy resistance in gastric cancer (GC). Despite efforts to target TAM recruitment or depletion, clinical efficacy remains limited. Consequently, the identification of targets that specifically inhibit or reprogram M2-polarized TAMs presents a promising therapeutic strategy.

**Objective:**

This study aims to identify a dual-function target in GC cells that drives both malignant phenotypes and M2 macrophage polarization, revealing its molecular mechanisms to provide novel therapeutic targets for selectivly targeting M2-polarized TAMs in GC.

**Methods:**

Transcriptomic and clinical data from GC and adjacent tissues were utilized to identify mRNAs associated with high M2 macrophage infiltration and poor prognosis. Single-cell sequencing elucidated cell types expressing the target gene. Transwell co-culture and exosome intervention experiments demonstrated its role in M2 polarization. Small RNA sequencing of exosomes, western blotting, and CoIP assays revealed the molecular mechanisms underlying exosome-mediated M2 polarization. Protein array, ChIP and dual-luciferase reporter assays clarified the molecular mechanisms by which the target gene regulated exosomal miRNA. In vivo validation was performed using xenograft tumor models.

**Results:**

*SERPINE1* was identified as a highly expressed mRNA in GC tissues and cells, significantly associated with advanced clinical stages, worse prognosis, and higher M2 macrophage infiltration in patients with GC. *SERPINE1* overexpression in GC cells promoted tumor growth and M2 macrophage polarization. *SERPINE1* facilitated the transfer of let-7 g-5p to macrophages via cancer-derived exosomes, inducing M2 polarization. Exosomal let-7 g-5p internalized by macrophages downregulated SOCS7 protein levels, disrupting its interaction with STAT3 and relieving the inhibition of STAT3 phosphorylation, thereby leading to STAT3 hyperactivation, which consequently drove M2 polarization. Additionally, in GC cells, elevated *SERPINE1* expression activated JAK2, enhancing STAT3 binding to the let-7 g-5p promoter and promoting its transcription, thereby increasing let-7 g-5p levels in exosomes.

**Conclusion:**

GC cell-derived *SERPINE1*, functioning as a primary driver of GC growth and TAM M2 polarization, promotes M2 polarization through the regulation of exosomal let-7 g-5p transfer via autocrine activation of the JAK2/STAT3 signaling pathway. These findings elucidate a novel mechanism of *SERPINE1*-induced M2 polarization and highlight SERPINE1 as a promising target for advancing immunotherapy and targeted treatments in GC.

**Supplementary Information:**

The online version contains supplementary material available at 10.1186/s13046-024-03269-4.

## Highlights

*SERPINE1* mediates the transfer of cancer-derived exosomal let-7 g-5p to promote macrophage M2 polarization.

Exosomal let-7 g-5p drives M2 polarization by downregulating SOCS7 and relieving its inhibition of STAT3 phosphorylation.

*SERPINE1* promotes the transcription of let-7 g-5p by activating the JAK2/STAT3 signaling pathway.

**Supplementary Information:**

The online version contains supplementary material available at 10.1186/s13046-024-03269-4.

## Introduction

M2-polarized tumor-associated macrophages (TAMs), recruited and driven by tumor-derived inflammatory cytokines and immunosuppressive metabolites, are predominant in many cancers, including gastric cancer (GC), where they promote tumor progression and contribute to chemotherapy resistance [1–3]. TAM depletion has been shown to enhance immunotherapy efficacy. Strategies such as CSF1/CSF1R inhibition or chimeric antigen receptor (CAR) T cells targeting specific TAM receptors can reduce TAM density, thereby improving chemotherapy sensitivity and antitumor effects [1, 4–8]. However, TAM depletion or recruitment blockade, either alone or in combination, has not yet yielded positive clinical outcomes [5]. This limitation may arise from the complexity of the tumor microenvironment (TME) and the heterogeneity of macrophage subsets, as well as the dual role of TAMs, where M1 TAMs exert cytotoxicity and phagocytosis, while M2 TAMs support tumor progression. Therefore, selectively targeting M2 TAMs or inhibiting M2 polarization to reshape the immunosuppressive TME, while preserving the antitumor functions of M1 TAMs, represents a potentially more effective therapeutic strategy.

Exosomes, as key mediators of intercellular communication, play a pivotal role in shaping the TME. Increasing evidence highlights that exosomes derived from GC cells significantly contribute to M2 macrophage polarization. For instance, GC cell-derived exosomal miR-92b-5p mediated by PLXNC1 promotes M2 polarization through inhibiting SOCS7-STAT3 interactions [9]. GC cell-derived exosomal circATP8A1 induces M2 polarization via the miR-1-3p/STAT6 axis [10]. Similarly, exosomal ELFN1-AS1 derived from GC cells facilitates macrophage recruitment and M2 polarization by regulating glycolysis through PKM in a HIF-1α-dependent manner [11]. GC cell-derived exosomal miR-541-5p drives M2 polarization through mediating the DUSP3/JAK2/STAT3 pathway [12]. GC cell-derived exosomal lncRNA HCG18 promotes macrophages M2 polarization by downregulating miR-875-3p to enhance KLF4 expression in macrophages [13]. GC cell-derived exosomal HMGB1 induces macrophages M2 polarization by inhibiting p50 transcriptional activity and inactivating the NF-κB pathway [14]. Moreover, GC cell-derived exosome-mediated M2 macrophage polarization significantly contributes to tumor progression and varying degrees of drug resistance [10–14]. Therefore, regulating the expression of certain substances in tumor-derived exosomes to inhibit M2 polarization or promote M1 type transformation may be an effective and promising multi-target therapeutic strategy for reshaping the immunosuppressive tumor microenvironment and enhancing antitumor efficacy.

Oncogene activation in tumor cells reshapes the tumor microenvironment and affects tumor progression and therapeutic response by promoting the secretion of cytokines and regulating the release of extracellular vesicles [15–17]. Therefore, identifying targets within tumor cells that simultaneously inhibit tumor progression and selectively suppress or reprogram M2-polarized TAMs represents offers a higher promising therapeutic strategy. This study utilized proprietary expression profile chip data from clinical GC tissues, transcriptomic data from public databases, relevant clinical information, and single-cell sequencing data from GC tissues to identify key genes that are highly expressed in GC cells and strongly associated with poor prognosis and elevated M2 macrophage infiltration. Subsequently, we clarified the role of these genes in mediating tumor-derived exosome-driven M2 macrophage polarization through a co-culture system of human monocytes and GC cells with gene silencing or overexpression, combined with exosome intervention assays. Additionally, Small RNA (sRNA) sequencing of tumor-derived exosomes identified oncogene-regulated miRNAs driving M2 polarization and elucidated their specific functions. Protein array analysis of gene-silenced GC cells further revealed the mechanisms by which oncogenes regulate exosomal miRNA expression. These findings provide important insights into the role of oncogenes in GC progression and mediating extracellular vesicle secretion, highlighting novel and promising therapeutic targets for both targeted and immunotherapy approaches in GC.

## Materials and methods

### Cells

GES-1 cells (BioChannel Biotech) were maintained in DMEM with high glucose. Human GC cell lines including MKN45, AGS, HGC27 (Pricella Biotech) and N87 (Genecarer) cells were cultured in RPMI 1640 medium (Gibco). THP1 cells (Ubigene) were grown in RPMI 1640 supplemented with 0.05 mM 2-mercaptoethanol (Sigma). All cells were incubated at 37 °C with 5% CO_2_ in medium containing 10% fetal bovine serum (FBS, Gibco) and 1% penicillin-streptomycin solution (Beyotime) to ensure optimal growth conditions.

### Clinical specimens

Clinical specimens were obtained from patients diagnosed with GC who underwent surgical resection at The Second Hospital of Lanzhou University. Fresh tissue samples for qRT-PCR and western blotting analyses were promptly frozen in liquid nitrogen. Tissue samples designated for immunofluorescence were fixed in 4% formaldehyde (Yuanye Biotech) to ensure optimal preservation for subsequent analysis.

### Human mRNA microarray analysis

Gene expression profile analysis of 16 pairs of GC and para-carcinoma tissues was performed using GeneChip™ Human Genome U133 Plus 2.0 microarray (Affymetrix, Santa Clara, USA). Chip scanning and data analysis were conducted by GeneChem Co., Ltd (Shanghai, China).

### Bioinformatics analysis

Transcriptomic and clinical data from The Cancer Genome Atlas (TCGA) Stomach Adenocarcinoma (STAD) cohort, available in GDC Data Portal (https://portal.gdc.cancer.gov/), were analyzed for differential gene expression, weighted gene co-expression network analysis (WGCNA) of M2 macrophage-related genes, immune cell infiltration estimation, univariate and multivariate Cox analysis, Kaplan-Meier (KM) survival analysis, correlation analysis of immune checkpoint expression, clinical characteristic evaluation, time-dependent ROC curve analysis, and Gene Set Enrichment Analysis (GSEA) to identify enriched signaling pathways between high and low gene expression groups. Tumor Immune Estimation Resource (TIMER) database (http://timer.cistrome.org/) was used for combined survival analysis of gene expression and M2 macrophage infiltration in GC patients. Single-cell sequencing data from GC tissues (GSE134520, GSE167297) in the Gene Expression Omnibus (GEO) database (https://www.ncbi.nlm.nih.gov/geo/) were used for cell clustering and gene expression analysis across different cell types. Transcriptome data from GC tissues (GSE118916, GSE33335 and GSE54129) in the GEO database were used for differential gene expression analysis. Transcription factors (TFs) regulating the miRNAs were predicted using TransmiR v2.0 (http://www.cuilab.cn/transmir), and JASPAR (http://jaspar.genereg.net) was employed to identify TF binding sites within the promoter regions. miRNA target gene prediction was performed using miRDB (http://mirdb.org/), miRWalk (http://mirwalk.umm.uni-heidelberg.de/), and miRTarBase (https://mirtarbase.cuhk.edu.cn/). KEGG pathway enrichment analysis of target genes was conducted utilizing the DAVID database (https://david.ncifcrf.gov/).

TCGA and GEO data were analyzed and visualized using R software (v. 4.2.1), utilizing R packages such as limma, clusterProfiler, pathview, GSEABase, WGCNA, timeROC, survival, survminer, complexheatmap, pheatmap, ggplot2, ggExtra, ggpubr and ggClusterNet. “CIBERSORT” and “Immunedeconv” were used to estimate the relative abundance of tumor-infiltrating immune cells (TIICs) in TCGA-STAD samples from normalized expression data.

### Lentiviral transfection

*SERPINE1*-lentiviral vector and control lentiviral vector were constructed and synthesized by VectorBuilder Biotech (Guangzhou, China) with lentivirus particle concentrations of approximately 6 × 10^8^ TU/ml. The targeting sequences were as follows: sh*SERPINE1*#1, 5′-GTGCCTGGTAGAAACTATTTC-3′; sh*SERPINE1*#2, 5′-AGACCAACAAGTTCAACTATA-3′; sh*SERPINE1*#3, 5′-TCTCTGCCCTCACCAACATTC-3′; and scramble shRNA (shNC) as a non-targeting negative control (NC), 5′-CCTAAGGTTAAGTCGCCCTCG-3′. Lentiviral particles at a multiplicity of infection (MOI) of 18, along with 5 µg/ml polybrene (VectorBuilder Biotech, Guangzhou, China), were introduced into 24-well plates containing cells seeded at a density of 6 × 10^4^ per well. Stable knockdown cell lines were generated by selecting in complete medium supplemented 2 µg/ml puromycin (VectorBuilder Biotech, Guangzhou, China) for two weeks.

### Transient transfection

*SERPINE1* overexpression plasmid and negative control plasmid were constructed and synthesized by VectorBuilder Biotech (Guangzhou, China). pcDNA3.1-EGFP-STAT3 plasmid and negative control plasmid were also obtained from VectorBuilder Biotech (Guangzhou, China). pCMV3-C-Myc-SOCS7 plasmid was constructed by Sino Biological Inc. (Beijing). Transfection complex was prepared by diluting 10 µl Lipo2000 and 4.0 µg plasmid in 250 µl Opti-MEM (Gibco) each, followed by gentle mixing. 3 × 10^5^ cells were seeded in 6-well plates, incubated with 1.5 ml Opti-MEM and 500 µl transfection complex for 6 h, and then cultured in complete medium for an additional 48 h.

AntagomiR negative control (NC, 5’-CAGUACUUUUGUGUAGUACAA-3’) and antagomiR-let-7 g-5p (5’-AACUGUACAAACUACUACCUCA-3’) were synthesized by GenePharma (Shanghai) and dissolved in 125 µl DEPC-treated water to prepare a 20 µM stock solution. THP1 cells (4 × 10^4^/well) were seeded in 24-well plates, incubated with a transfection complex (20 pmol antagomiR, 1 µl Lipo2000 and 100 µl pti-MEM) and 400 µl Opti-MEM for 6 h, followed by replacement with PMA-containing medium and cultured for 24 h.

### Immunofluorescence (IF)

Tissue sections were heated at 58 °C for 2 h, then deparaffinized in xylene and rehydrated through a graded ethanol series. Cells were washed, fixed with 4% paraformaldehyde, and permeabilized using 0.2% Triton X-100 (Solarbio). Antigen retrieval was performed using sodium citrate buffer (pH 6.0, 98 °C), followed by goat serum blocking for 1 h. Sections were incubated with PAI-1 (rabbit, 1:200, Immunoway), CD163 (mouse, 1:200, Immunoway), CD206 (mouse, 1:200, Proteintech), F4/80 (rabbit, 1:200, Bioss), iNOS (rabbit, 1:200, Bioss), Arg1 (rabbit, 1:200, Proteintech), STAT3 (rabbit, 1:200, Bioss) antibodies overnight at 4 °C, reactivated, stained with Cy3-conjugated goat anti-rabbit IgG (Abcam) and Alexa Fluor 488-conjugated goat anti-mouse IgG (Abcam) for 30 min, counterstained with DAPI, and imaged using fluorescence microscopy (IX51, Olympus) for ImageJ analysis.

### Flow cytometry assay

THP-1 cells were differentiated into macrophages using 150 ng/mL phorbol 12-myristate 13-acetate (PMA, Sigma) for 24 h and subsequently co-cultured with cancer-derived exosomes or GC cells in 6-well plates with 0.4-µm membranes for 72 h. Harvested macrophages were converted into single-cell suspensions, stained with Elab Fluor 488 anti-human CD68 (Mouse, 1:20, ElabScience) and APC anti-human CD206 (Mouse, 1:20, ElabScience) antibodies, and analyzed for CD68^+^CD206^+^ populations by flow cytometry (Accuri C6, BD).

### Quantitative real-time polymerase chain reaction (qRT-PCR)

Total RNA was extracted using TRIzol reagent (Invitrogen) and reverse transcribed into cDNA using the Hifair III 1st Strand cDNA Synthesis Kit (Yeasen). Quantitative PCR (qPCR) was performed on a Real-Time PCR System (7500, Applied Biosystems) using Hieff UNICON Universal Blue QPCR SYBR Green Master Mix (Yeasen). Total miRNA was isolated with the miRNeasy Mini Kit (Qiagen) and reverse transcribed into first-strand cDNA using the Mir-X miRNA First Strand Synthesis Kit (Takara). qPCR was conducted using the Mir-X miRNA qRT-PCR TB Green Kit (Takara). Relative quantification (2^−ΔΔCT^) was normalized to GAPDH and U6 snRNA. Primer sequences are provided in the Supplementary Materials.

### Western blotting

Tissues, cells and exosomes were lysed using RIPA buffer (Solarbio) supplemented with 1% PMSF (Sigma), and the lysates were centrifuged at 12,000 × g for 15 min at 4 °C. Protein concentrations were quantified via a BCA assay (Solarbio). Proteins were then separated by SDS-PAGE and transferred onto PVDF membranes (Millipore). After blocking with 5% fat-free milk, membranes were incubated overnight with primary antibodies (Supplementary Materials). Chemiluminescent substrates (Affinity) were used for detection, and blots were visualized using SH-Compact523 Chemiluminescence Gel Imaging System (Shenhua).

### Cell viability

Cell viability was assessed using a CCK8 kit (PUMOKE), and absorbance at 450 nm was measured with a microplate reader (iMark, Bio-Rad).

### EdU incorporation assay

Cell proliferation was evaluated using EdU staining, following the manufacturer’s instructions (Solarbio).

### Colony formation

A colony formation assay was conducted using 0.3% soft agar. In each well of a 6-well plate, 400–800 cells were seeded and cultured for two weeks. Colonies were then stained with 0.5% crystal violet.

### Exosome isolation and identification

2 × 10⁵ GC cells silencing or overexpressing SERPINE1 were seeded into T75 culture flasks and cultured to approximately 80% confluence. The medium was then replaced with serum-free media (Umibio) and incubated for an additional 48 h. Subsequently, equal volume of conditioned medium was collected for exosome isolation by ultracentrifugation (XPN-100, 32Ti rotor, Beckman) and re-suspended in 200 µl of cold 1 × PBS. Exosome concentration and size were analyzed via BCA protein assay (Solarbao), nanoparticle tracking analysis (ZetaView PMX110, Germany), while their morphology and size were confirmed by TEM (Hitachi-7500, Japan). Protein markers were detected using western blotting. Dil-labeled exosomes (Yeasen) internalized by macrophages were visualized using confocal laser scanning microscopy (CLSM; SP8, Leica).

### Migration and invasion assays

Transwell inserts (8 μm, Corning) were employed to assess cell migration and invasion. 500 µl complete medium was added to the lower chamber of a 24-well plate, and 100 µl of cell suspension (approximately 2 × 10^4^ cells) was placed in the upper chamber for 48 h. Invasion assays were conducted by coating the upper chamber membranes with 100 µl Matrigel Matrix (Corning), followed by the same steps as the migration assay. Invading cells were methanol-fixed, stained with crystal violet, and imaged using an IX51 microscope (Olympus).

### Transmission electron microscopy (TEM)

Exosome solution was diluted 1:10 with 1 × PBS, and 10 µl was applied onto a piece of parafilm, followed by placement of a 200-mesh Formvar-carbon copper grid for 20 min. The grid was fixed in 2.5% glutaraldehyde for 5 min, washed in deionized water, stained with 4% uranyl acetate for 10 min, stained with methylcellulose-UA on ice for 10 min and observed by Hitachi-7500 TEM.

### Small RNA sequencing (sRNA seq) of exosomes

sRNA sequencing was performed by RiboBio Biotech (Guangzhou). Total RNA was isolated from six exosome samples (three from the *SERPINE1* knockdown group and three from the control group) using the miRNeasy Micro Kit (QIAGEN). RNA quality and integrity were verified with an ND-1000 Spectrophotometer (NanoDrop Technologies) and a Bioanalyzer 2100 (Agilent). A sequencing library was constructed from 1 µg of total RNA per sample using the NEBNext Multiplex Small RNA Library Prep Set for Illumina (NEB), and sequencing was conducted on the Illumina HiSeq2500 platform. Differential expression of exosomal miRNAs was evaluated using reads per million (RPM) and DESeq2 (V1.26.0).

### Protein microarray analysis

Protein microarray analysis was conducted using the CSP100 Plus Microarray (HWayen), which immobilizes highly specific antibodies involving 16 signaling pathways. Each antibody had six technical replicates, scanned using an Agilent SureScan Dx Microarray Scanner, and image intensities were analyzed with GenePix Pro v6.0 software (Axon).

### Xenograft experiments

Specific pathogen-free (SPF)-grade BALB/c-nu mice (male, 5 weeks old) were obtained from Chengdu Yaokang Biotech Co. Ltd and kept under SPF conditions. Twelve mice were randomly divided into two groups: MKN45/AGS cells transfected with scrambled shRNA or sh*SERPINE1*#3 (*n* = 3/group). Each mouse was subcutaneously injected with 5 × 10⁶ cells mixed with 50% Matrigel (Corning). Tumor volumes were measured weekly. After 42 days, mice were sacrificed, and tumors were excised, weighed, and divided for western blotting and immunofluorescence analyses.

### Co-immunoprecipitation (Co-IP)

Macrophages induced by PMA were exposed to exosomes isolated from GC cells and transfected with pCMV3-C-Myc-SOCS7 plasmids as before, with or without antagomir-92b-5p. Co-IP was conducted using the Pierce Co-IP Kit (Thermo Scientific). Cell lysates were pre-cleared with Protein A/G beads to remove non-specific proteins, with 20% reserved as input control. The remaining lysates were incubated overnight at 4 °C with anti-STAT3 (Rabbit, 1:50, Cell Signaling), anti-Myc (Rabbit, 1:200, Beyotime) or IgG (Rabbit, 1:50, Abcam), followed by Protein A/G bead capture. After washing to remove unbound proteins, complexes were eluted, denatured, and analyzed via western blotting.

### Fluorescence in situ hybridization (FISH)

FISH analysis was performed using the miRNA FISH Kit (GenePharma). A Cy3-labeled hsa-let-7 g-5p probe (GenePharma) was synthesized as follows: 5′ Cy3-AACTGTACAAACTACTACCTCA-3′ and provided by GenePharma. Briefly, paraffin Sect. (5 μm) were incubated at 60 °C for 30 min, deparaffinized with xylene, and rehydrated through graded ethanol. Proteinase K digestion (20 min at 37 °C) and denaturation (8 min at 78 °C) followed. Cells were fixed with 4% formaldehyde for 15 min at room temperature, washed with PBS, and hybridized with denatured probes at 37 °C for 12 h. Nuclei were counterstained with DAPI (GenePharma), and images were captured using an Olympus BX51 fluorescence microscope.

### Chromatin immunoprecipitation (ChIP)

ChIP assays were performed using a ChIP Kit (Beyotime) in accordance with established protocols. MKN45 cells were transfected with pcDNA3.1-EGFP-STAT3 plasmids as before. Cells at 24 h post-transfection were crosslinked with 1% formaldehyde at 37 °C for 10 min, followed by quenching with 125 mM glycine at room temperature for 5 min. Cells were subsequently collected, lysed, and subjected to sonication to fragment the DNA into 200–750 bp segments, confirmed by agarose gel electrophoresis. A 10% aliquot of each chromatin complex was reserved as an input control. IP was performed using anti-pSTAT3 (Rabbit, 1:50, Cell Signaling Technology) or Rabbit IgG (1:50, Abcam) antibodies, with Protein A/G Agarose/Salmon Sperm DNA to capture immune complexes associated with the let-7 g-5p promoter (site position: -1666 to -1483). After sequential washes, qPCR was employed to quantify the immunoprecipitated DNA, with ΔΔCt values calculated according to previous literature [9]. Specific primers used are provided in the Supplementary Materials.

### Dual-luciferase reporter gene assay

The potential STAT3-binding sites on the let-7 g-5p promoter were identified using JASPAR (http://jaspar.genereg.net/). The promoter region (-1692 to -1420) of let-7 g-5p was synthesized and inserted into the pRP[Pro]-hRluc/Puro-Luciferase reporter plasmids including negative control, wild-type and mutant-type (VectorBuilder), which were transfected into MKN45 cells. Luciferase activity was evaluated using the Dual-Luciferase Reporter Assay System (Promega). Relative luciferase activity was calculated following the method described in the previous literature [9].

### Statistical analysis

Data analysis and visualization were conducted using SPSS 26.0 (IBM, Chicago, IL, USA) and GraphPad Prism v9 (GraphPad Software, San Diego, CA, USA). Categorical variables were analyzed with a chi-squared test, and correlations were determined using Spearman’s correlation test. Nonparametric data were analyzed using the Mann-Whitney U test for two-variable comparisons and the Kruskal-Wallis test for multiple variables. Normally distributed continuous data were analyzed using Student’s t-tests or paired t-tests for two-group comparisons and one-way ANOVA with Tukey’s post hoc test for multiple comparisons. All experiments were independently replicated at least three times. Statistical significance was set at *p* < 0.05 and shown as * *p* < 0.05, ** *p* < 0.01, *** *p* < 0.001, **** *p* < 0.0001, ns (not significant) *p* > 0.05.

## Results

### *SERPINE1* is identified as a key factor associated with poor prognosis and a driver of M2 macrophage infiltration in GC

**Fig. 1:**
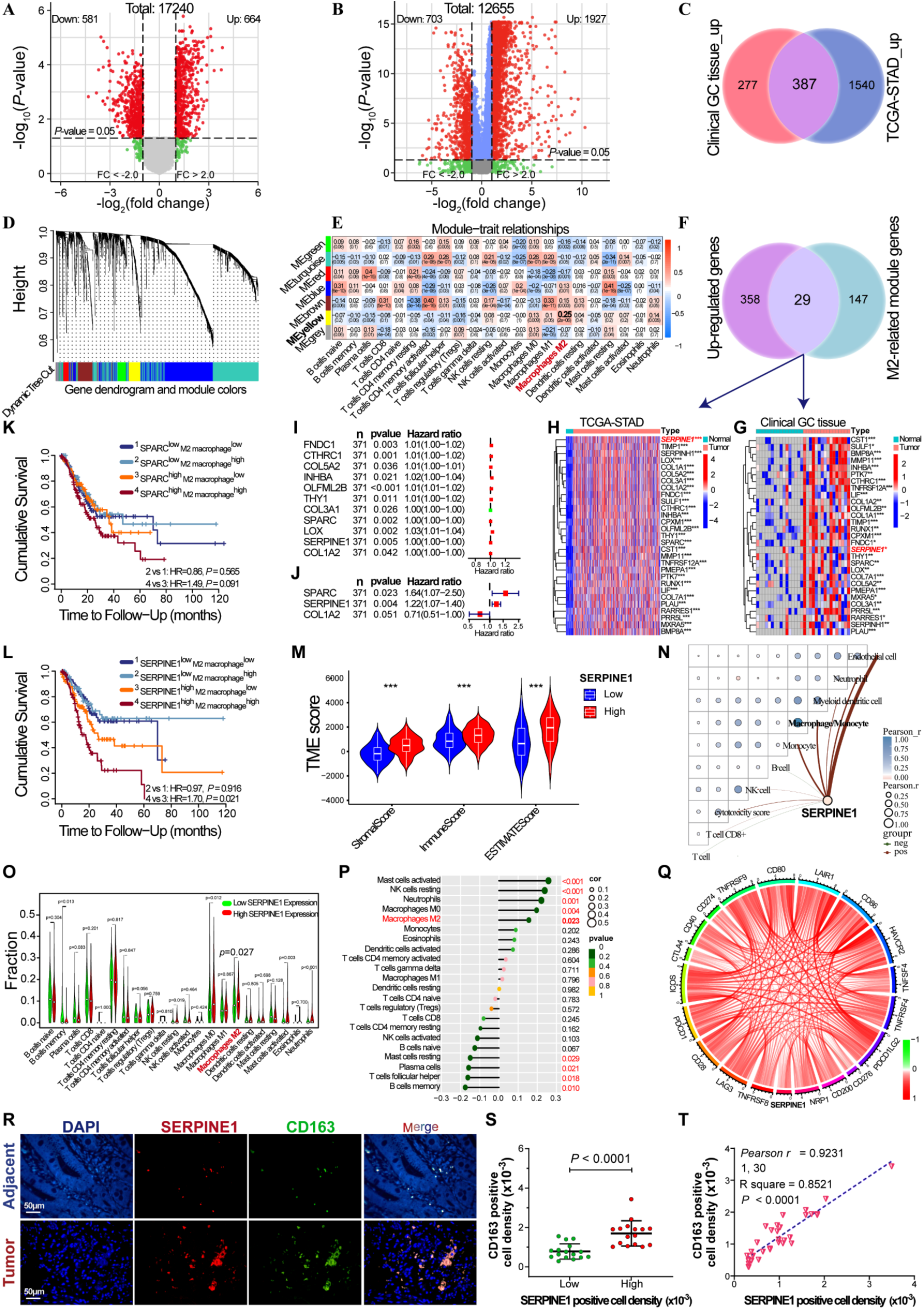
Screening of genes associated with M2 macrophages and prognosis of GC. Volcano plots of differential mRNA expression in 16 GC patients (**A**) and TCGA-STAD cohort (**B**); red dots indicate upregulated genes, and green dots indicate downregulated genes. (**C**) Venn diagram of upregulated mRNAs in 16 GC patients and the TCGA-STAD cohort. (**D**) WGCNA cluster dendrogram and module assignment using a dynamic tree-cutting algorithm. (**E**) Correlation between module genes and immune cell infiltration. The abscissa represents different types of immune cell infiltration and the ordinate represents different modules. Each rectangle displayed the Pearson correlation coefficient. (**F**) Venn diagram of the upregulated mRNAs and M2-related yellow module mRNAs. (**G** and **H**) Heatmap of M2-related module-mRNA expression in 16 GC patients and the TCGA-STAD cohort. (**I** and **J**) Forest plots of univariate and multivariate Cox regression analysis of the M2-related module mRNAs. Kaplan-Meier cumulative survival curves for the combined analysis of *SPARC* (**K**) or *SERPINE1* (**L**) expression and M2 macrophage infiltration in GC. (**M**) Differential analysis of immune stromal components between high- and low-*SERPINE1* expression groups. (**N**) Correlation analysis of *SERPINE1* expression and immune cells. (**O**) Differential analysis of immune cell infiltration between high- and low-*SERPINE1* expression groups. (**P**) Correlation analysis of *SERPINE1* expression and immune cell infiltration. (**Q**) Correlation analysis of *SERPINE1* expression with immune checkpoint expression. (**R**) Immunofluorescence analysis of CD163 and *SERPINE1* expression in 32 pairs of GC and non-GC tissue. (**S**) Difference of CD163-positive cell density between high and low *SERPINE1*-positive cell density groups. (**T**) Correlation analysis of CD163-positive and *SERPINE1*-positive cell densities in 32 GC tissues

Human mRNA microarray analysis of 16 pairs of GC and para-carcinoma tissues identified 664 upregulated and 581 downregulated mRNAs (Fig. 1A). Similarly, differential mRNA analysis of the TCGA-STAD cohort revealed 1927 upregulated and 703 downregulated mRNAs across 375 GC and 32 para-carcinoma tissues (Fig. 1B), with 387 mRNAs co-upregulated (Fig. 1C). CIBERSORT immune cell infiltration distribution and 2630 differential mRNA expression data in TCGA-STAD cohort were used for WGCNA analysis (Fig. 1D-E). A scale-free network was constructed at an optimal soft-threshold (*β* = 4) when R^2^ exceeded 0.9, generating seven modules (Fig. 1D), with 176 mRNAs in the yellow module identified as M2 macrophage-related genes. Subsequently, a total of 29 mRNAs were identified as being associated with M2 macrophages and prognosis (Fig. 1F), and their expression validated in both the 16 pairs of GC and para-carcinoma tissues (Fig. 1G) and the TCGA-STAD cohort (Fig. 1H). Univariate and multivariate Cox regression analysis of 29 mRNAs in the TCGA-STAD cohort (Fig. 1I-J) showed that *SPARC* and *SERPINE1* were independent prognostic factors (*p* < 0.05). Kaplan-Meier survival curves (Figure S1A-C) indicated that high *SPARC*, *SERPINE1*, and *COL1A2* expression correlated with shorter overall survival (OS) in the TCGA-STAD cohort. The TIMER database indicated a shorter OS in GC patients with high *SERPINE1* expression and high M2 macrophage infiltration (Fig. 1K-L). Kaplan-Meier survival analysis of 380 GC patients undergoing surgery alone from merged GEO datasets (GSE14120, GSE15459, GSE22377, GSE29272, GSE51105, GSE62254) and patients from GSE62254 and GSE22377 further confirmed that high SERPINE1 expression is associated with shorter OS (Figure S1D-E).

StromalScore and immuneScore estimated by R package “estimate” significantly increased in GC tissues with high-*SERPINE1* expression (Fig. 1M). An “Immunedeconv” algorithm determined the proportion of 10 types of cells in each GC tissues of TCGA-STAD corhort and showed that *SERPINE1* expression was significantly positively correlated with macrophage (Fig. 1N). The CIBERSORT deconvolution algorithm determined the proportion of 22 types of immune-infiltrating cells in each GC tissue of TCGA-STAD cohort and showed a higher proportion of M2 macrophage infiltration in the high-*SERPINE1* expression group (Fig. 1O) and a significant positive correlation between *SERPINE1* expression and M2 macrophages (Fig. 1P). Moreover, *SERPINE1* expression positively correlated with the expression of 19 immune checkpoints (Fig. 1Q), displaying immunosuppressive features. Immunofluorescence assays of 32 GC tissues (Fig. 1R) showed higher CD163-positive cell density in the high *SERPINE1*-positive cell density group (Fig. 1S) and a significant positive correlation between CD163- and *SERPINE1*- positive cell densities (Fig. 1T).

### Elevated *SERPINE1* promotes M2 macrophage polarization in GC

**Fig. 2:**
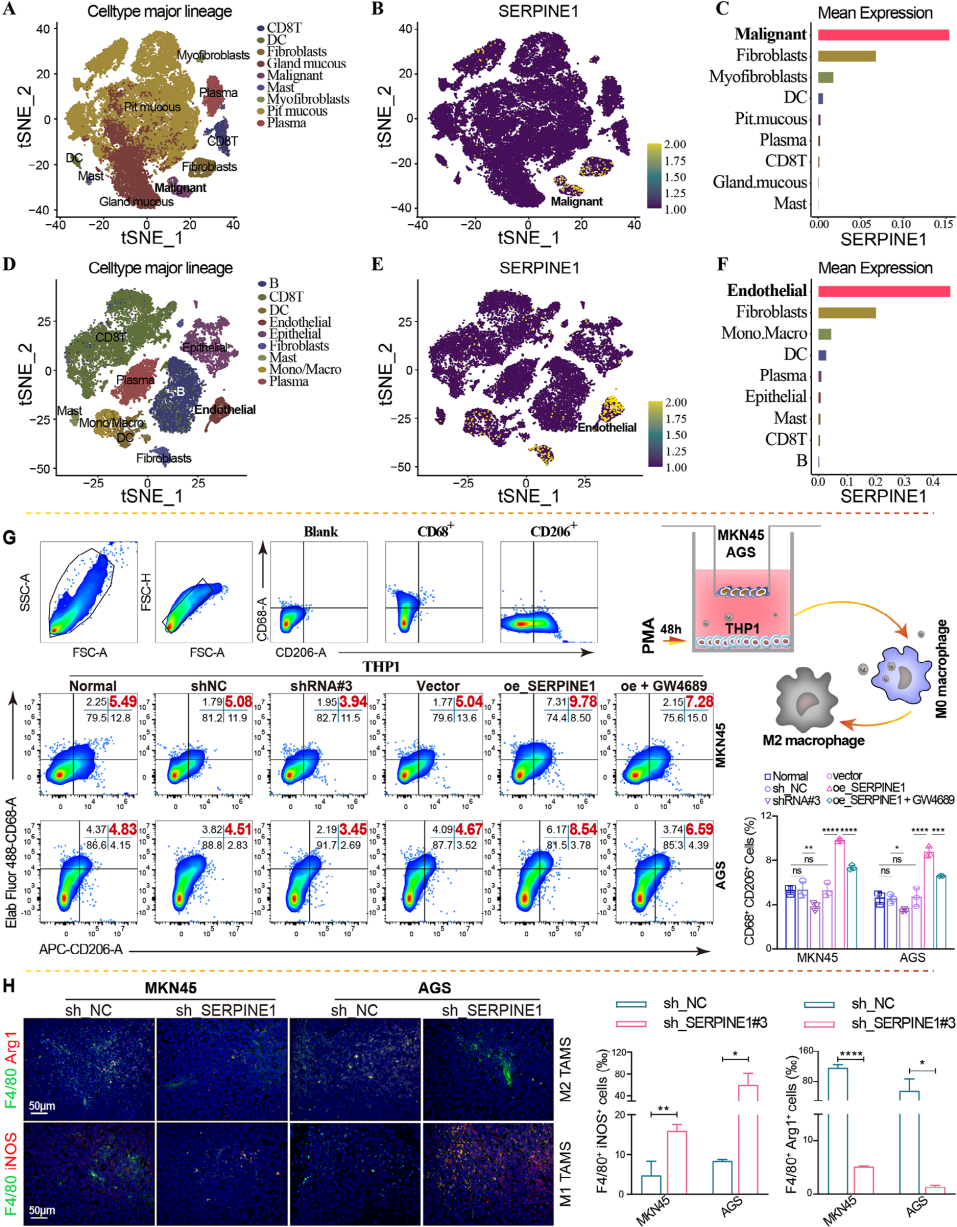
High *SERPINE1* expression in GC cells promotes macrophage M2 polarization. tSNE visualization of nine single-cell clusters partitioned by unsupervised cluster analysis, *SERPINE1* expression of each single-cell, *and SERPINE1* expression abundance of different single-cell clusters in the GSE134520 (**A**–**C**) and GSE167297 (**D**–**F**) datasets. (**G**) Flow cytometry analysis of the proportion of CD68^+^CD206^+^ macrophages in a Transwell co-culture system, with MKN45 and AGS cells overexpressing (oe_*SERPINE1*) or silencing *SERPINE1* (shRNA#3 or sh_*SERPINE1*#3) in the upper chamber, and THP1 cells treated with PMA in the lower chamber. (**H**) Immunofluorescence staining of xenograft tumor tissues. Comparison of the proportion of M1 or M2 macrophage infiltration. Green indicates F4/80. Red indicates iNOS or Arg1 expression

Single-cell sequencing results from the GSE134520 (Fig. 2A-C) and GSE167297 (Fig. 2D–F) datasets revealed that *SERPINE1* was highly expressed in GC cells. To further investigate the effect of SERPINE1 expression on macrophage M2 polarization, SERPINE1 silencing and overexpression plasmids were constructed for stable lentiviral transfection and transient transfection, respectively. qRT-PCR (Fig. S1G–J) and western blotting (Figure S1K) verified that SERPINE1 expression was remarkably downregulated in the silenced group and upregulated in the overexpression group. A Transwell co-culture system further showed that *SERPINE1* overexpression promoted macrophage M2 polarization, and this effect was reversed by GW4689 (an exosome inhibitor) (Fig. 2G). Additionally, immunofluorescence staining with F4/80 (a macrophage marker) and iNOS (an M1 polarization marker)/Arg1 (an M2 polarization marker) was used to investigate the impact of *SERPINE1* on M2 macrophage infiltration in xenograft tumors, revealing decreased F4/80^+^Arg1^+^ cells (M2 TAMs) and increased F4/80^+^iNOS^+^ cells (M1 TAMs) in xenograft tumors of GC cells with stably silenced *SERPINE1* (Fig. 2H).

### *SERPINE1* is inversely correlated with GC progression

**Fig. 3:**
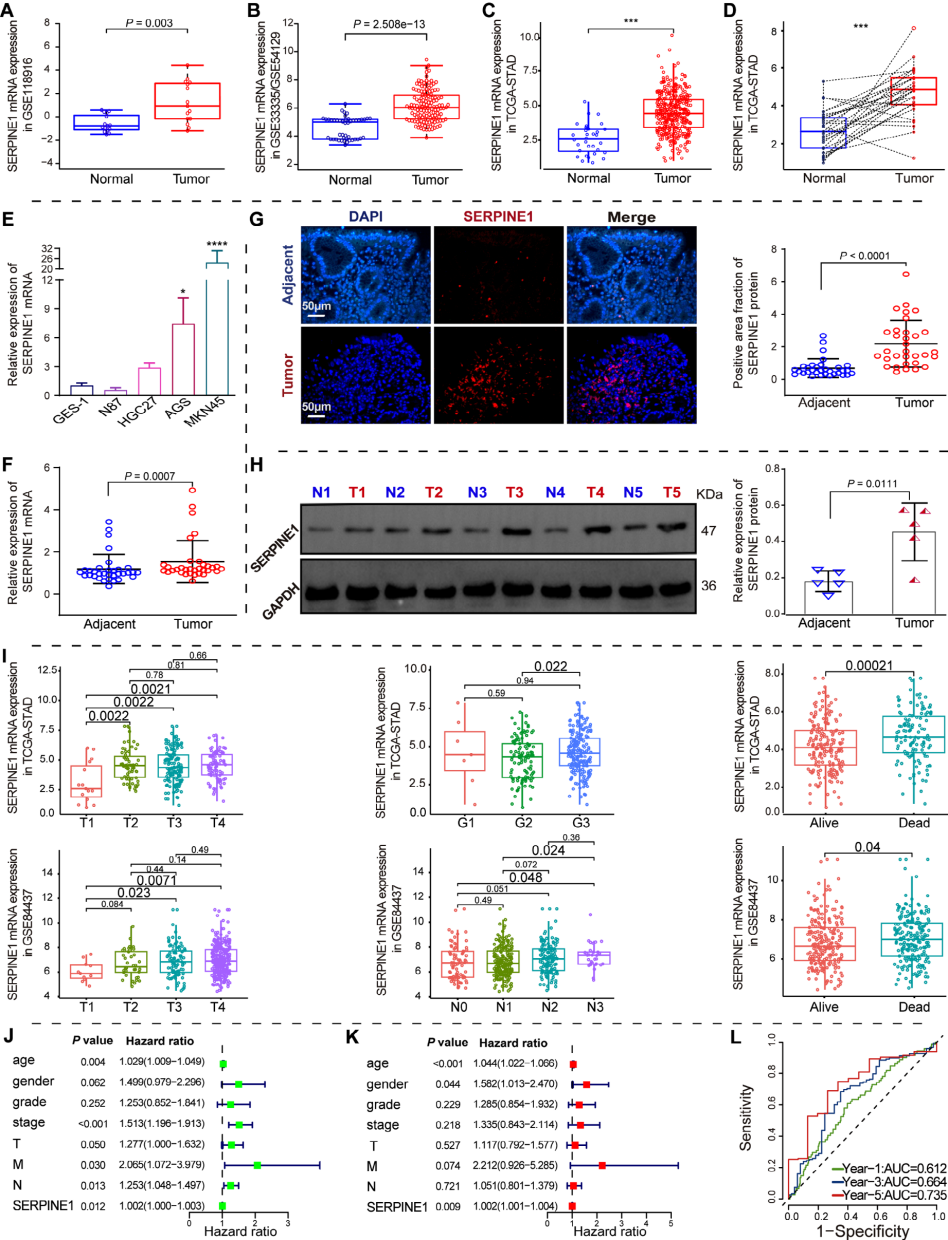
Differential expression and prognosis analysis of *SERPINE1*. (**A**–**D**) Differential expression of *SERPINE1* mRNA in merged GSE33335/GSE54129 (A), GSE118916 (**B**), and TCGA-STAD (**C** and **D**) cohorts. (**E**) Differential *SERPINE1* mRNA expression in GC and GES-1 cells. qRT-PCR (**F**), immunofluorescence (**G**), and western blotting (**H**) analysis of *SERPINE1* mRNA and protein expression in GC and non-GC tissues. (**I**) Differential expression of *SERPINE1* in GC patients with different clinical stages and survival statuses in TCGA-STAD cohorts and GSE84437 dataset. (**J**–**K**) Forest plots of univariate and multivariate Cox regression analysis of GC prognosis. (**L**) Time-dependent ROC curves for OS at different time points to assess the predictive ability of *SERPINE1* mRNA expression

*SERPINE1* mRNA expression was significantly upregulated in GC tissues, as confirmed by the GSE118916 dataset (Fig. 3A), the merged GSE33335 and GSE54129 datasets (Fig. 3B), and the TCGA-STAD cohort (Fig. 3C-D). qRT-PCR analysis revealed higher *SERPINE1* expression in AGS and MKN45 cells (*p* < 0.05) (Fig. 3E). A higher level of *SERPINE1* mRNA and protein expression was confirmed in GC tissues from 33 patients using qRT-PCR (Fig. 3F, *p* < 0.001), immunofluorescence (Fig. 3G, *p* < 0.0001), and western blotting analysis (Fig. 3H, *p* < 0.05). Higher *SERPINE1* expression was observed in GC patients with higher T, N and G stages (*p* < 0.05) and in deceased GC patients (*p* < 0.05) (Fig. 3I). Univariate and multivariate Cox regression analysis of the TCGA-STAD cohort identified *SERPINE1* mRNA level as an independent predictor (Fig. 3J-K). A ROC curve analysis estimated the predictive value of *SERPINE1* mRNA level in TCGA-STAD cohort. The AUC values for 1-, 3- and 5-years OS for patients with GC were 0.612, 0.664, and 0.735, respectively (Fig. 3L). Correlation analysis showed *that SERPINE1* mRNA expression correlated with T stage (Table 1).

**Table 1:** Demographic and clinicopathological variables of GC patients

Pathological feature	*n*	SERPINE1		*P*-value
		Low expression	High expression	
Total	33	16	17	
Gender				0.934568
Male	27	13	14	
Female	6	3	3	
Age				0.737139
<65	26	13	13	
≥65	7	3	4	
T *				0.027868
T1-T2	4	4	0	
T3-T4	29	12	17	
N				0.575942
NO	5	3	2	
YES	28	13	15	
M				0.324534
M0	32	16	16	
M1	1	0	1	
Stage				0.118989
I–II	14	9	5	
III-IV	19	7	12	
Lauren classification				0.174235
Intestinal	6	3	3	
Diffuse	17	8	9	
Mixed	10	5	5	
Histological				0.170656
Well differentiated	2	2	0	
Moderate differentiated	17	7	10	
Poorly differentiated	14	7	7	

### *SERPINE1* confers GC progression

**Fig. 4:**
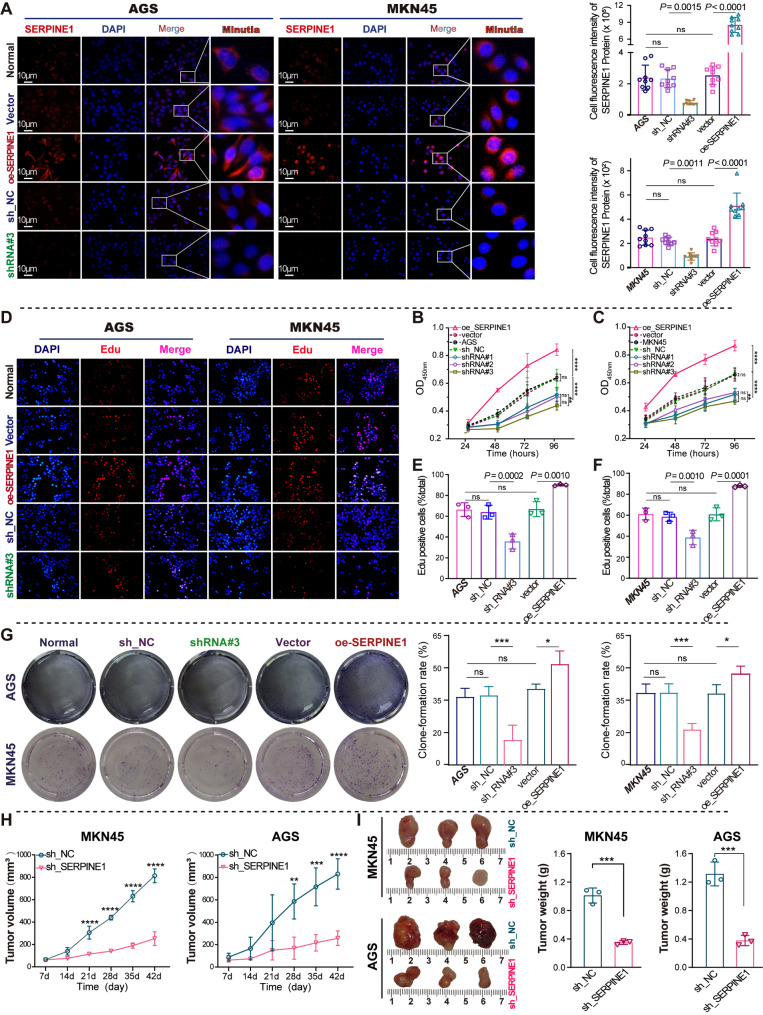
*SERPINE1* promotes GC cell proliferation in vitro and in vivo. (**A**) Immunofluorescence analysis of *SERPINE1* protein cellular localization and expression in GC cells with *SERPINE1* silencing and overexpression. (**B**–**G**) Cell proliferation assays for GC cells with *SERPINE1* silencing (sh_*SERPINE1*#3) and overexpression (oe_*SERPINE1*): CCK8 (**B** and **C**), EdU (**D**–**F**), and colony formation assay (**G**). (**H** and **I**) Nude mice were observed 42 days after subcutaneous injection of MKN45/AGS cells with either silenced (sh*SERPINE1*#3) or non-silenced *SERPINE1* (shNC). (**H**) Growth curves of xenograft tumor volumes. (**I**) Comparison of tumor weights between sh*SERPINE1*#3 and shNC groups

To elucidate the role of SERPINE1 in GC progression, its impact on GC cell proliferation was analyzed through both in vitro and in vivo studies. Immunofluorescence further confirmed the cellular localization and expression of *SERPINE1* protein in silenced and overexpressed cells, which was mainly localized in the cytoplasm (Fig. 4A), and the protein expression was consistent with the results of qRT-PCR (Fig. S1G–J) and western blotting (Fig. S1K). *SERPINE1* knockdown led to a notable decrease in the proliferation of GC cells, as determined by CCK8 (Fig. 4B-C), EdU (Fig. 4D–F), and colony formation assays (Fig. 4G). Overexpression significantly enhances GC cell proliferation. A reduced growth rate (Fig. 4H) and lower tumor weight (Fig. 4I) were observed in xenograft tumor models subcutaneously injected with GC cells with silenced *SERPINE1*.

### *SERPINE1*-mediated GC cell-derived exosomes promote macrophage M2 polarization

**Fig. 5:**
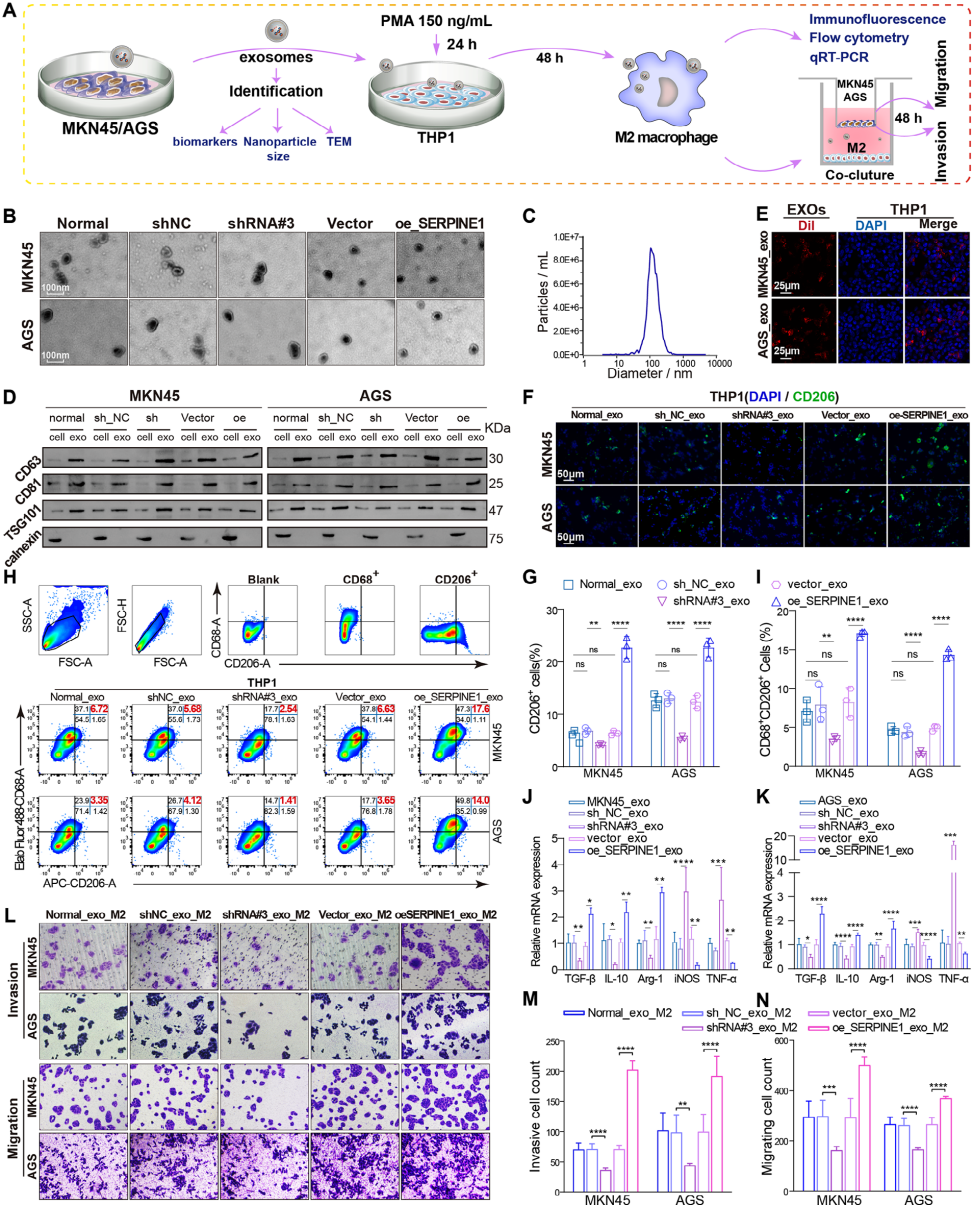
*SERPINE1*-mediated gastric cancer-derived exosomes facilitate the polarization of THP1 cells into M2 macrophages. (**A**) Schematic representation of the extraction and identification of exosomes and the induction of macrophage polarization. Transmission electron microscopy (**B**), nanoparticle tracking analysis (**C**), and western blotting (**D**) were used to identify the morphology, particle size, and markers of exosomes. (**E**) Confocal laser scanning microscopy detected Dil-labeled exosomes (red) internalized by DAPI-labeled macrophages (blue). (**F**–**G**) Immunofluorescence analysis of the proportion of CD206^+^ cells in THP1 cells treated with exosomes. (**H**–**I**) Flow cytometry analysis of the proportion of CD68^+^CD206^+^ cells in THP1 cells treated with exosomes. (**J**–**K**) qRT-PCR analysis of M1 markers (iNOS and TNF-α) and M2 markers (TGF-β, IL-10, and Arg-1) in THP1 cells treated with exosomes. (**L**–**N**) Transwell migration and invasion assays of GC cells (upper chamber) co-cultured with macrophages (lower chamber) ingesting exosomes

Exosomes were enriched by ultracentrifugation from equal volumes of serum-free, exosome-depleted culture medium derived from GC cells silencing or overexpressing *SERPINE1* at the same initial cell densities, and used in subsequent experiments to determine the effects of cancer-derived exosomes on M2 polarization (Fig. 5A). TEM showed typical “saucer shape” particles with a size of approximately 100 nm (Fig. 5B). NTA analysis revealed homogeneous particle sizes with a mean diameter of 137.6 nm (Fig. 5C). Exosome concentrations were quantified using the BCA assay with a BSA standard curve (Supplementary Fig. 2), and the results indicated that concentrations across all groups ranged from 66 to 69 µg/mL, showing consistent levels and high quality (Supplementary Table1). Western blotting analysis revealed the presence of CD63, CD81, and TSG101 exosomal markers as well as the absence of calnexin (Fig. 5D). CLSM showed that exosomes were successfully internalized into PMA-induced THP1 cells (Fig. 5E). A higher proportion of CD206^+^ and CD68^+^CD206^+^ macrophages was observed in PMA-induced THP1 cells ingesting exosomes derived from GC cells overexpressing *SERPINE1* by immunofluorescence analysis (Fig. 5F-G) and flow cytometry analysis (Fig. 5H-I).

qRT-PCR analysis of classical M1 and M2 markers revealed decreased M1 markers and increased M2 markers in macrophages ingesting exosomes derived from GC cells overexpressing *SERPINE1* (Fig. 5J-K). A Transwell co-culture system showed that macrophages ingesting exosomes derived from GC cells overexpressing *SERPINE1* promoted GC cell invasion and migration (Fig. 5L-N). As presented above, exosomes derived from GC cells overexpressing *SERPINE1* significantly enhance M2 polarization, resulting in greater pro-migration and pro-invasive potential.

### *SERPINE1*-mediated exosomal let-7 g-5p derived from GC cells polarizes macrophages into M2 macrophages by promoting STAT3 hyperphosphorylation

**Fig. 6:**
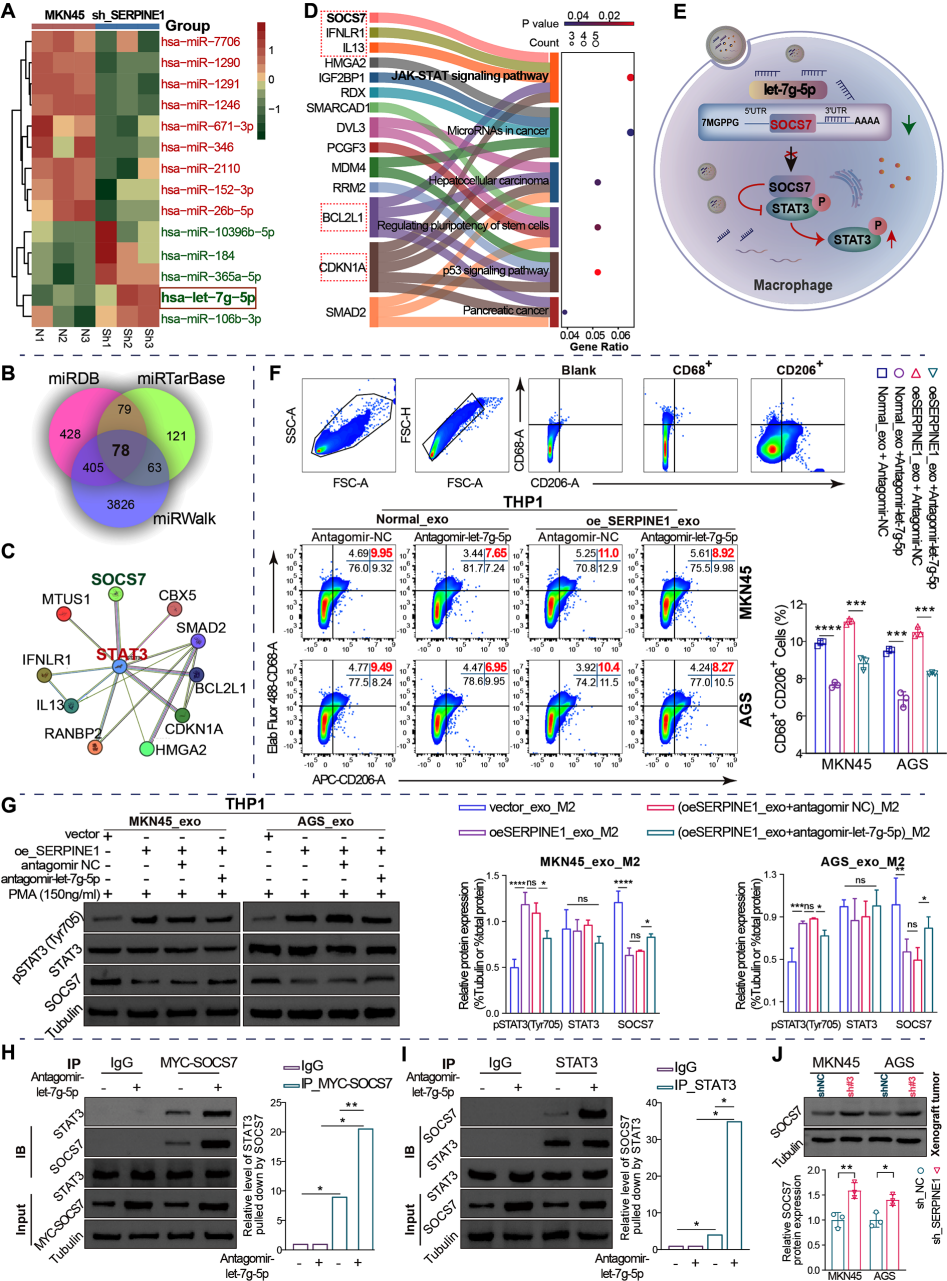
*SERPINE1*-mediated GC-derived exosomal let-7 g-5p facilitates macrophage M2 polarization through STAT3 hyperphosphorylation resulting from inhibition of SOCS7 interactions with STAT3. (**A**) Differential miRNA analysis of exosomes derived from MKN45 cells with stably silenced *SERPINE1* and normal MKN45 cells using sRNA-Seq. N, normal group. sh, stably silenced *SERPINE1*. (**B**) Venn diagram of target genes predicted by miRDB, miRWalk, and miRTarBase for let-7 g-5p. (**C**) Network of target genes that interact with STAT3. (**D**) KEGG pathway analysis of the 78 target genes of let-7 g-5p using DAVID. (**E**) Schematic representation: exosomal let-7 g-5p ingested by macrophages inhibits SOCS7 interaction with STAT3, resulting in STAT3 hyperphosphorylation. (**F**) Flow cytometric assay of the impact of let-7 g-5p on M2 polarization induced by exosomes derived from GC cells. (**G**) Western blotting analysis for the levels of SOCS7 protein and STAT3 phosphorylation in macrophages treated with exosomes and antagomir-let-7 g-5p. (**H** and **I**) Endogenous CoIP assay for SOCS7 and STAT3 in macrophages ingesting exosomes derived from normal MKN45 cells. (**J**) Western blotting analysis of SOCS7 protein levels in xenograft tumors

An sRNA-Seq analysis of exosomal miRNA profiles from MKN45 cells stably silencing *SERPINE1* compared with normal MKN45 cells identified 14 differentially expressed miRNAs with log_2_(foldchange) > 1.2 and *p* < 0.05, including 9 upregulated miRNAs and 5 downregulated miRNAs (Fig. 6A). Exosomal let-7 g-5p was the most statistically significant downregulated miRNA (*p* = 0.002).

According to the intersection of miRDB, miRWalk, and miRTarBase, 78 potential targets of let-7 g-5p were predicted (Fig. 6B). Signal transducer and activator of transcription family 3 (STAT3), a member of the STAT family, plays a key role in M2 polarization by being activated through phosphorylation, dimerization, and nuclear translocation [18, 19]. Protein interaction analysis of the 78 targets and STAT3 revealed that 10 target genes interacted with STAT3 using the STRING database (Fig. 6C). KEGG pathway enrichment analysis of 78 potential targets showed that 5 proteins interacting with STAT3, including SOCS7, IFNLR1, IL13, BCL2L1, and CDKN1A, were enriched in the Janus kinase (JAK)-STAT signaling pathway (Fig. 6D). It has been reported that STAT3 phosphorylation and nuclear translocation can be negatively regulated by suppressors of cytokine signaling (SOCS) 7 [20, 21]. Thus, exosomal let-7 g-5p may bind to the 3′ untranslated region (UTR) of SOCS7 to suppress SOCS7 protein synthesis through mRNA degradation or translation repression, resulting in the alleviation of the dephosphorylation and phosphorylation inhibition of STAT3 mediated by SOCS7 and subsequent STAT3 activation in macrophages (Fig. 6E).

Flow cytometry analysis demonstrated that antagomir-let-7 g-5p reversed M2 polarization induced by exosomes derived from GC cells overexpressing *SERPINE1* (Fig. 6F). However, it is unclear whether SOCS7 interacts with STAT3 to regulate M2 polarization. In this study, decreased SOCS7 protein levels and increased STAT3 phosphorylation levels were observed in macrophages ingesting exosomes derived from GC cells overexpressing *SERPINE1*, and antagomir-let-7 g-5p reversed this effect (Fig. 6G). It appears that let-7 g-5p negatively regulated SOCS7 protein expression and promoted STAT3 phosphorylation. Subsequently, a Co-IP experiment demonstrated that SOCS7 and STAT3 co-precipitated in GC cell-derived exosome-taking macrophages, further confirming that SOCS7 physically interacts with STAT3. Additionally, the interaction was enhanced by antagomir-let-7 g-5p (Fig. 6H-I). Finally, western blotting analysis demonstrated a significant elevation in SOCS7 levels following *SERPINE1* silencing in vivo (Fig. 6J).

These findings indicate that let-7 g-5p negatively regulates SOCS7 protein levels and consequently decreased SOCS7 interaction with STAT3, leading to STAT3 hyperphosphorylation to facilitate M2 polarization.

### *SERPINE1* positively regulates exosomal let-7 g-5p through the JAK2/STAT3 signaling pathway

**Fig. 7:**
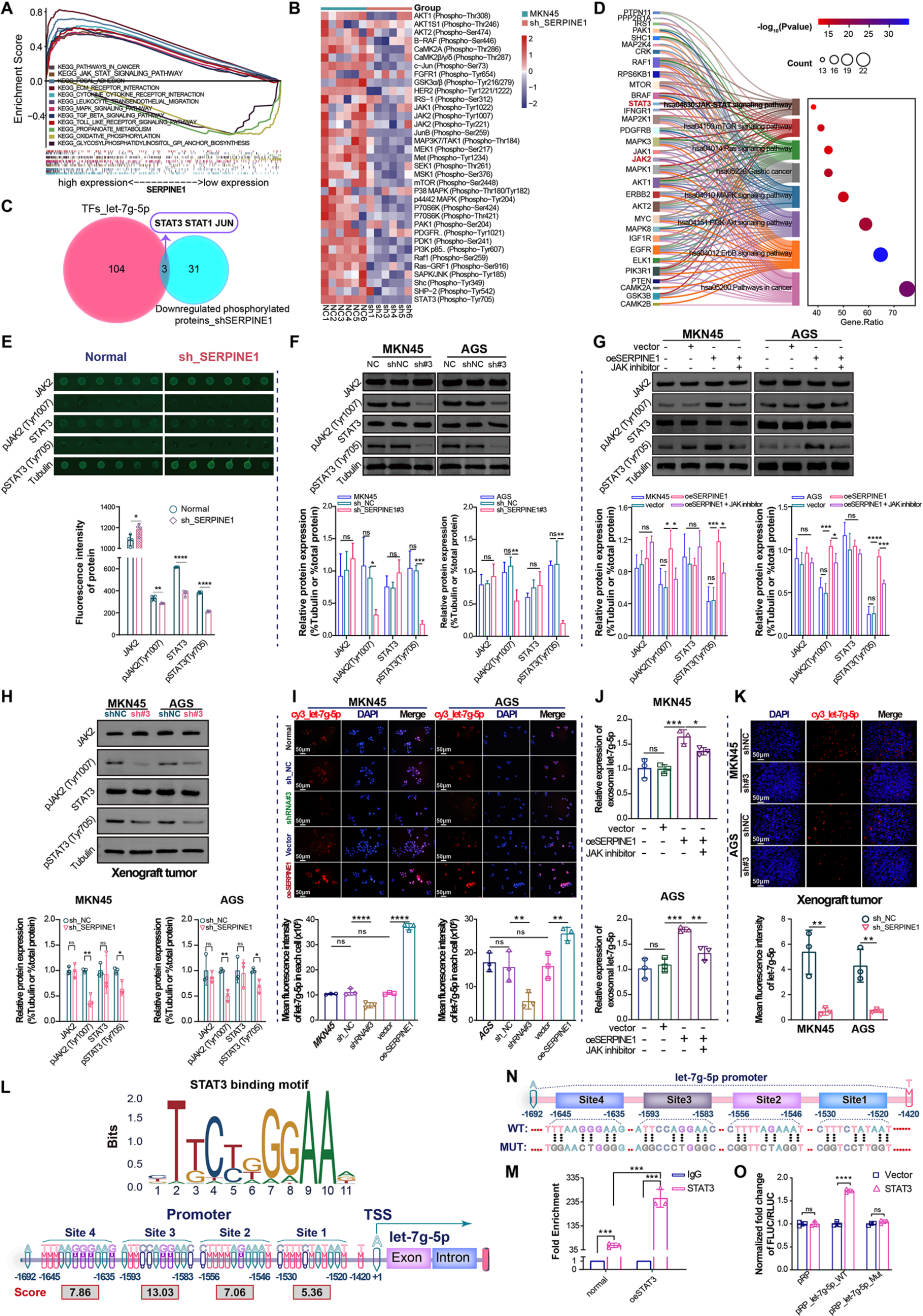
*SERPINE1* promotes exosomal let-7 g-5p expression through the JAK2/STAT3 pathway. (**A**) GSEA was conducted for *SERPINE1* co-expressed genes using GSEA (version 4.1.0). (**B**) Heatmap of 35 phosphorylation sites of 34 STAT3 upstream proteins determined by the median fluorescent intensity of the protein array normalized using Grubb’s algorithm. (**C**) Venn diagram of 34 downregulated phosphorylated proteins in the silenced *SERPINE1* group and 107 TFs targeting let-7 g-5p. (**D**) Bubble plot combined with Sankey diagram of enriched KEGG pathways for 32 of the 34 STAT3 upstream proteins. (**E**) Statistical analysis of normalized phospho- and nonphospho-fluorescent protein spots in protein arrays. Western blotting analysis of total protein and phosphorylation levels of JAK2/STAT3 in GC cells silencing *SERPINE1* (**F**), overexpressing *SERPINE1* or treated with a JAK inhibitor (**G**). (**H**) Western blotting analysis of JAK2/STAT3 and SOCS7 in xenograft tumors. (**I**) Representative FISH images and comparison of let-7 g-5p expression in GC cells. (**J**) qRT-PCR analysis of exosomal let-7 g-5p expression in GC cells. (**K**) Representative FISH images and comparison of let-7 g-5p expression in xenograft tissues. (**L**) STAT3-binding motif and sites in the let-7 g-5p promoter region predicted using the JASPR database. (**M**) ChIP-qPCR assay demonstrated that STAT3 interacted with the let-7 g-5p promoter (site position: -1666~-1483). (**N**) Dual-luciferase reporter gene assay for the let-7 g-5p promoter region (position: -1692~-1420). (**O**) Luciferase activity of wt- and mut- let-7 g-5p promoter in the presence of vector or oeSTAT3. Mut, mutated-type. WT, wild-type. Vector, negative control plasmid. oeSTAT3, STAT3 overexpression plasmid

To confirm how *SERPINE1* regulates exosomal let-7 g-5p expression, GSEA, protein microarray, and TF prediction were used sequentially. According to gene set enrichment analysis (GSEA), *SERPINE1* expression was significantly correlated with the activation of the JAK-STAT pathway (Fig. 7A). Protein array analysis identified 34 phosphorylated proteins that were downregulated in MKN45 cells with stably silenced SERPINE1 (Fig. 7B). Among these, STAT3, STAT1, and JUN were screened by intersecting 34 downregulated phosphorylated proteins with 107 TFs targeting let-7 g-5p predicted by the TransmiR v2.0 database (Fig. 7C).

STAT3 serves as a central hub for multiple oncogenic signaling pathways and oncogenes, contributing to GC progression and chemotherapy resistance [22, 23]. KEGG pathway enrichment analysis showed that JAK2 and STAT3 were simultaneously enriched in JAK-STAT pathway (Fig. 7D). JAK2 and STAT3 were phosphorylated to a lesser extent in the *SERPINE1*-silenced group based on the fluorescent intensity of the protein array (Fig. 7E). Therefore, it is highly likely that *SERPINE1* activates JAK2-STAT3 signaling to transcriptionally regulate let-7 g-5p.

Additionally, western blotting analysis confirmed that pJAK2 (Tyr1007) and pSTAT3 (Tyr705) levels decreased in GC cells silencing *SERPINE1* (Fig. 7F), and increased in GC cells overexpressing *SERPINE1* (Fig. 7G). This suggests that high *SERPINE1* expression activates the JAK2-STAT3 signaling pathway. A JAK inhibitor (Fedratinib, MedChemExpress) blocked the activation of JAK2/STAT3 stimulated by high *SERPINE1* expression (Fig. 7G). Western blotting analysis revealed significantly decreased phosphorylation levels of JAK2 and STAT3 in xenograft tumors silencing *SERPINE1* (Fig. 7H), providing further evidence of the influence of *SERPINE1* on the activation of JAK2-STAT3 signaling pathway in vivo.

FISH analysis of let-7 g-5p expression in GC cells showed higher expression in GC cells overexpressing *SERPINE1* and lower expression in GC cells silencing *SERPINE1* (Fig. 7I). qRT-PCR also revealed higher let-7 g-5p expression in equal volumes of exosomes derived from GC cells overexpressing *SERPINE1*, which was partially reversed by the JAK inhibitor (Fig. 7J). Consistent results were observed in FISH analysis of xenograft tumor tissues, where *SERPINE1*-silenced xenografts showed reduced let-7 g-5p expression (Fig. 7K). Overall, *SERPINE1* contributed to GC cell-derived exosomal let-7 g-5p expression by activating JAK2/STAT3.

However, whether STAT3 directly regulates let-7 g-5p transcription remains unknown. According to the JASPR database, STAT3 can bind directly to the promoter of let-7 g-5p (Fig. 7L). Subsequent ChIP-qPCR analysis indicated robust binding affinity of STAT3 to the let-7 g-5p promoter (Fig. 7M). In addition, dual-luciferase reporter gene assays revealed that STAT3 enhanced the activity of the wild-type let-7 g-5p promoter (Fig. 7N-O).

## Discussion

Oncogene activation in tumor cells plays a crucial role in driving the transfer of tumor-derived exosomes to macrophages, which induces M2 polarization and contributes to tumor progression and therapy resistance [14–16]. In this study, transcriptomic and single-cell sequencing analyses from GC tissues identified *SERPINE1* as a gene highly expressed in GC cells, closely associated with poor prognosis and elevated M2 macrophage infiltration. Subsequent experiments revealed that highly expressed *SERPINE1* promotes GC growth and upregulates let-7 g-5p transcription in GC cells through the activation of JAK2/STAT3 signaling pathway. Furthermore, *SERPINE1*-mediated transfer of exosomal let-7 g-5p disrupts the SOCS7-STAT3 interaction in macrophages, resulting in STAT3 hyperactivation and driving macrophage M2 polarization.

*Serine protease inhibitor family E member 1* (*SERPINE1*), encoding plasminogen activator inhibitor-1 (PAI-1, a 45-kDa glycoprotein), is highly expressed in various tumors and serves as a cancer-promoting factor by facilitating tumor cell proliferation, migration, invasion, and angiogenesis in GC [24–26]. Recent studies have demonstrated that *SERPINE1* is significantly overexpressed in GC and holds considerable potential as a prognostic marker [27–29]. In this study, *SERPINE1* was found to be significantly upregulated in GC through high-throughput mRNA microarray analysis of 16 paired GC and adjacent tissues, combined with differential mRNA expression analysis of the TCGA-STAD cohort, consistent with previous reports. Further correlation analysis of clinical characteristics, immune cell infiltration, and prognosis revealed that high *SERPINE1* expression was strongly associated with higher M2 macrophage infiltration, advanced clinical stage, and poor prognosis in GC. These findings suggest that *SERPINE1* may serve as a potential marker for GC subtyping, immunophenotyping, and prognosis prediction. Additionally, this study revealed that silencing *SERPINE1* significantly inhibits GC growth both in vitro and in vivo, aligning with previous findings [24]. Collectively, these results highlight *SERPINE1* as an oncogene and suggest that targeting it may provide a novel therapeutic strategy to impede GC progression.

*SERPINE1*/PAI-1 has garnered increasing attention for its association with M2 macrophage infiltration and immunotherapeutic response in the GC [26, 27, 29, 30]. However, the specific functions and mechanisms of *SERPINE1* within the GC microenvironment remain unclear. Although reports on *SERPINE1* promoting M2 polarization in macrophages are limited, its role and mechanism in driving this process are being increasingly recognized. A study on esophageal squamous cell carcinoma demonstrated that PAI-1 derived from cancer-associated fibroblasts (CAFs) promotes macrophage migration by activating Akt and Erk 1/2 through interaction with low-density lipoprotein receptor-related protein 1 (LRP1), an endocytic receptor on macrophage surfaces [31]. Similarly, research on fibrosarcoma and lung cancer cells showed that cancer cell-derived PAI-1 stimulates macrophage migration via its LRP1 binding domain and induces M2 polarization by activating the p38MAPK/NF-kB signaling pathway and the IL6/STAT3 autocrine loop through its C-terminal urokinase plasminogen activator (uPA) binding domain [32]. Consequently, it is clear that PAI-1, derived from cancer cells or CAFs in a paracrine manner, plays a crucial role in the regulation of macrophage recruitment and M2 polarization.

In this study, single-cell sequencing analysis revealed that *SERPINE1* was primarily expressed in GC cells. It is hypothesized that *SERPINE1* promotes macrophage M2 polarization in GC through a paracrine secretion mechanism. Supporting this hypothesis, *SERPINE1* overexpression significantly enhanced M2 polarization in human monocytes co-cultured with GC cells. Further investigation using an exosome inhibitor, which blocks exosome production in GC cells, reversed the M2 polarization induced by *SERPINE1* overexpression, indicating that beyond the paracrine pathway, *SERPINE1* also promotes macrophage M2 polarization through tumor-derived exosomes. To further validate this finding, exosomes were isolated from GC cells with either silenced or overexpressed *SERPINE1* and subsequently applied to human monocytes, demonstrating that exosomes derived from GC cells with overexpressed *SERPINE1* significantly promoted M2 polarization, while those from GC cells with silenced *SERPINE1* exhibited the opposite effect. This finding not only confirms that *SERPINE1* overexpression in GC cells promotes M2 macrophage polarization, but also provides a novel perspective beyond previously recognized paracrine mechanisms, highlighting that SERPINE1 mediates the transfer of tumor-derived exosomes to enhance M2 polarization.

Exosomes, as pivotal mediators of intercellular communication within TME, play an essential role in mediating cellular interactions. Tumor-derived exosomes transfer proteins, lipids, and miRNAs to macrophages, reprogramming their gene expression and metabolic pathways and driving macrophages from the anti-tumor M1 phenotype toward the pro-tumor M2 phenotype, thereby promoting tumor growth and progression [33–35]. In this study, exosome sRNA sequencing analysis revealed that let-7 g-5p was the predominant miRNA carried within *SERPINE1*-mediated exosomes. Subsequent exploration showed that inhibition of let-7 g-5p significantly reversed macrophage M2 polarization induced by GC cell-derived exosomes, including those from *SERPINE1*-overexpressing cells. This finding aligns with previous studies showing that tumor-derived exosomes reprogram immune cells by delivering oncogenic factors, such as miRNAs, which influence macrophage behavior and promote immune evasion and tumor growth [35, 36]. However, research on the function of let-7 g-5p remains limited, and its regulatory mechanisms in macrophage M2 polarization represent an important question that warrants further investigation.

STAT3 plays a pivotal role in M2 polarization through its phosphorylation, dimerization, and nuclear translocation [18, 19]. In this study, SOCS7 was identified as an interacting protein of STAT3 and a target of let-7 g-5p. SOCS7 is known to negatively regulate STAT3 phosphorylation and nuclear translocation [20, 21]. Thus, exosomal let-7 g-5p is speculated to bind to the 3’UTR of SOCS7 mRNA, promoting its degradation or inhibiting translation, thereby relieving SOCS7-mediated inhibition of STAT3 phosphorylation, leading to excessive STAT3 activation and M2 polarization in macrophages. In this study, SOCS7 protein levels decreased, whereas STAT3 phosphorylation increased in macrophages exposed to exosomes derived from *SERPINE1*-overexpressing cells, and this effect was reversed by antagomir-let-7 g-5p. Subsequent protein interaction assays further confirmed the physical interaction between SOCS7 and STAT3 in macrophages exposed to GC cell-derived exosomes, and let-7 g-5p inhibition enhanced this interaction. This alteration in SOCS7 expression was validated in vivo. The results suggest that *SERPINE1*-mediated cancer-derived exosomal let-7 g-5p downregulates SOCS7 protein levels, reducing its interaction with STAT3, which leads to STAT3 hyperphosphorylation and promotes M2 polarization. Additionally, previous studies have shown that STAT5 phosphorylation inhibits macrophage M1 polarization and promotes its shift toward the M2 phenotype [37, 38]. SOCS7 has been reported to interact with STAT5, inhibiting its phosphorylation and nuclear translocation [39]. Therefore, it is possible that exosomal let-7 g-5p may promote M2 polarization by reducing the inhibitory effect of SOCS7 on STAT5. Moreover, MAP3K1 has been identified as a predicted target of let-7 g-5p and is essential for activating NF-κB and p38/JNK signaling pathways, which are critical for macrophage M1 polarization [40]. Inhibition of MAP3K1 has been shown to promote the transition from M1 to M2 macrophages [41]. Thus, exosomal let-7 g-5p may target MAP3K1 to disrupt M1 polarization-related signaling and promote the conversion of M1 macrophages to the M2 phenotype. These findings offer valuable insights into the role of exosomal let-7 g-5p in shaping an immunosuppressive tumor microenvironment, providing a critical direction for future research.

However, this may represent only a small part of *SERPINE1*’s role in facilitating macrophage M2 polarization by mediating the transfer of exosomal miRNAs. Among the downregulated exosomal miRNAs derived from *SERPINE1*-silenced GC cells, miR-365a-5p has been reported to promote macrophage M2 polarization by inhibiting the TLR2/MyD88/NF-κB signaling pathway in osteoarthritis [42]. Similarly, miR-106b-3p decreased significantly in ferroptotic cardiomyocyte-derived exosomes, where it promoted macrophage M1 polarization by activating the Wnt signaling pathway [43]. These findings suggest that, in addition to let-7 g-5p, *SERPINE1* silencing may inhibit M2 polarization and promote M1 polarization by downregulating miR-365a-5p and miR-106b-3p. Conversely, among the upregulated miRNAs, miR-26b-5p, miR-671-3p, miR-152-3p, miR-1246, miR-1290, miR-346, and miR-1291 were predicted to target HMGA2, a known activator of STAT3 transcription and a driver of macrophage recruitment [44]. Notably, a separate study has shown that downregulation of miR-1291 by osteosarcoma-derived exosomal ELFN1-AS1 promotes macrophage M2 polarization via upregulating CREB1 [45]. These observations highlight a potential mechanism through which *SERPINE1* silencing reduces M2 macrophage infiltration by inhibiting HMGA2 and upregulating miR-1291. Additionally, miR-671-3p inhibition has been shown to promote macrophage M2 polarization via the KLF12/AKT/c-myc signaling pathway in pancreatic cancer [46]. Thus, its upregulation following *SERPINE1* silencing may further disrupt M2 polarization. Collectively, these findings suggest that *SERPINE1* silencing modulates macrophage polarization and recruitment through multiple miRNA-mediated signaling pathways, providing critical insights into its regulatory role in M2 polarization.

At this moment, another critical question regarding the regulatory mechanism of *SERPINE1* on let-7 g-5p expression remains unanswered and urgently needs clarification. Based on transcription factor prediction for let-7 g-5p and protein microarray analysis in *SERPINE1*-silenced GC cells, we hypothesized that *SERPINE1* transcriptionally regulate the expression of let-7 g-5p by activating JAK2 to promote STAT3 phosphorylation and nuclear translocation. Our results confirmed that silencing *SERPINE1* inhibited both JAK2 and STAT3 phosphorylation in vivo and in vitro. Additionally, subsequent cell experiments revealed that *SERPINE1* overexpression elevated exosomal let-7 g-5p levels, an effect counteracted by a JAK inhibitor. Further analysis revealed that STAT3 could directly bind to the let-7 g-5p promoter, enhancing its transcriptional activity. These findings suggest that *SERPINE1* overexpression promotes let-7 g-5p transcription by activating the JAK2/STAT3 signaling pathway, thereby elevating exosomal let-7 g-5p levels.

Given that JAK2/STAT3 is frequently activated in various malignancies, including GC, where it drives cell proliferation, migration, invasion, and shapes the immunosuppressive microenvironment [47, 48], the discovery that *SERPINE1* activates the JAK2/STAT3 pathway not only reveals the regulatory mechanism of exosomal let-7 g-5p, but also provides a potential explanation for the role of *SERPINE1* in promoting GC cell proliferation. This finding adds a new layer of understanding by showing that *SERPINE1* regulates and mediates the transfer of exosomal miRNAs through the JAK2/STAT3 signaling axis to favor tumor progression. However, whether *SERPINE1* activates JAK2 through cytoplasmic protein interactions or via autocrine signaling through LRP1 and the C-terminal uPA-binding domain on the GC cell surface remains unclear and warrants further study.

## Conclusions

**Fig. 8:**
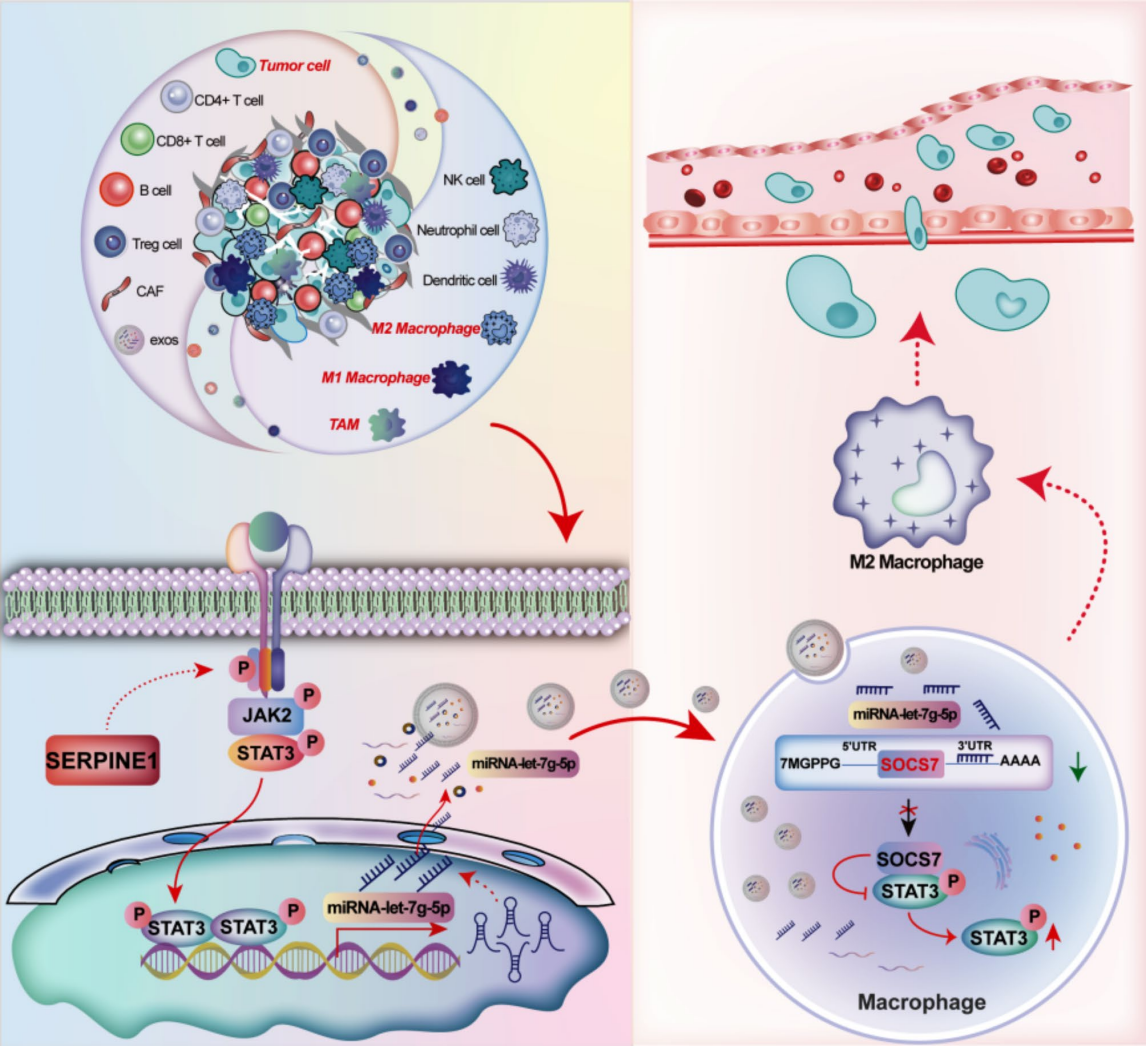
Graphical summarization of the molecular mechanism of *SERPINE1*-mediated Gastric cancer-derived exosomes promoting macrophage M2 polarization

In conclusion, this study demonstrates the dual role of *SERPINE1* in promoting GC cell proliferation and driving TAM M2 polarization through both autocrine signaling and exosome-mediated communication. As shown in Fig. 8, this study highlights that *SERPINE1* transcriptionally regulates exosomal let-7 g-5p levels derived from GC cells through the JAK2/STAT3 signaling pathway. By transferring exosomal let-7 g-5p to macrophages, *SERPINE1* disrupts the SOCS7-STAT3 interaction, thereby lifting SOCS7-mediated suppression of STAT3 phosphorylation, leading to STAT3 hyperactivation and subsequent macrophage M2 polarization. This study establishes *SERPINE1* as a key regulator driving macrophage M2 polarization and immune suppression through exosome-mediated miRNA transfer. Targeting *SERPINE1*, whether by inhibiting JAK2/STAT3 signaling in GC cells, blocking exosomal let-7 g-5p translocation, or disrupting STAT3 activation in macrophages, presents a novel therapeutic strategy to reprogram the immunosuppressive microenvironment, potentially transforming GC treatment through enhanced anti-tumor immunity.

## Electronic supplementary material

Below is the link to the electronic supplementary material.

Supplementary Material 1

## Data Availability

Data produced in this study are available within the article and its accompanying supplementary files.

## Abbreviations

CAFs: Cancer-associated fibroblasts
CAR: Chimeric antigen receptor
ChIP: Chromatin immunoprecipitation
Co-IP: Co-Immunoprecipitation
exo: Exosome
FBS: Fetal bovine serum
FISH: Fluorescence in situ hybridization
GC: Gastric cancer
GEO: Gene Expression Omnibus
GSEA: Gene Set Enrichment Analysis
IF: Immunofluorescence
JAK: Janus kinase
KM: Kaplan-Meier
LRP1: Low-density lipoprotein receptor-related protein 1
NC: Negative control
qPCR: Quantitative PCR
qRT-PCR: Quantitative real-time polymerase chain reaction
SERPINE1: Serine protease inhibitor family E member 1
shNC: Scramble shRNA
SOCS7: Suppressors of cytokine signaling 7
SPF: Specific pathogen-free
sRNA seq: Small RNA sequencing
STAD: Stomach Adenocarcinoma
STAT3: Signal transducer and activator of transcription family 3
TAMs: Tumor-associated macrophages
TCGA: The Cancer Genome Atlas
TEM: Transmission electron microscopy
TF: Transcription factor
TIICs: Tumor-infiltrating immune cells
TIMER: Tumor Immune Estimation Resource
TME: Tumor microenvironment
uPA: Urokinase plasminogen activator
UTR: Untranslated region
WGCNA: Weighted gene co-expression network analysis
