# Supplementary material for: Gastric cancer-derived exosomal let-7 g-5p mediated by SERPINE1 promotes macrophage M2 polarization and gastric cancer progression
Source: J Exp Clin Cancer Res. 2025 Jan 2;44:2. doi: 10.1186/s13046-024-03269-4 (PMC11694445; doi:10.1186/s13046-024-03269-4)
Supplement: Supplementary file 1 — Supplementary Material 1 [file 13046_2024_3269_MOESM1_ESM.docx]

**Supplementary Materials**

**
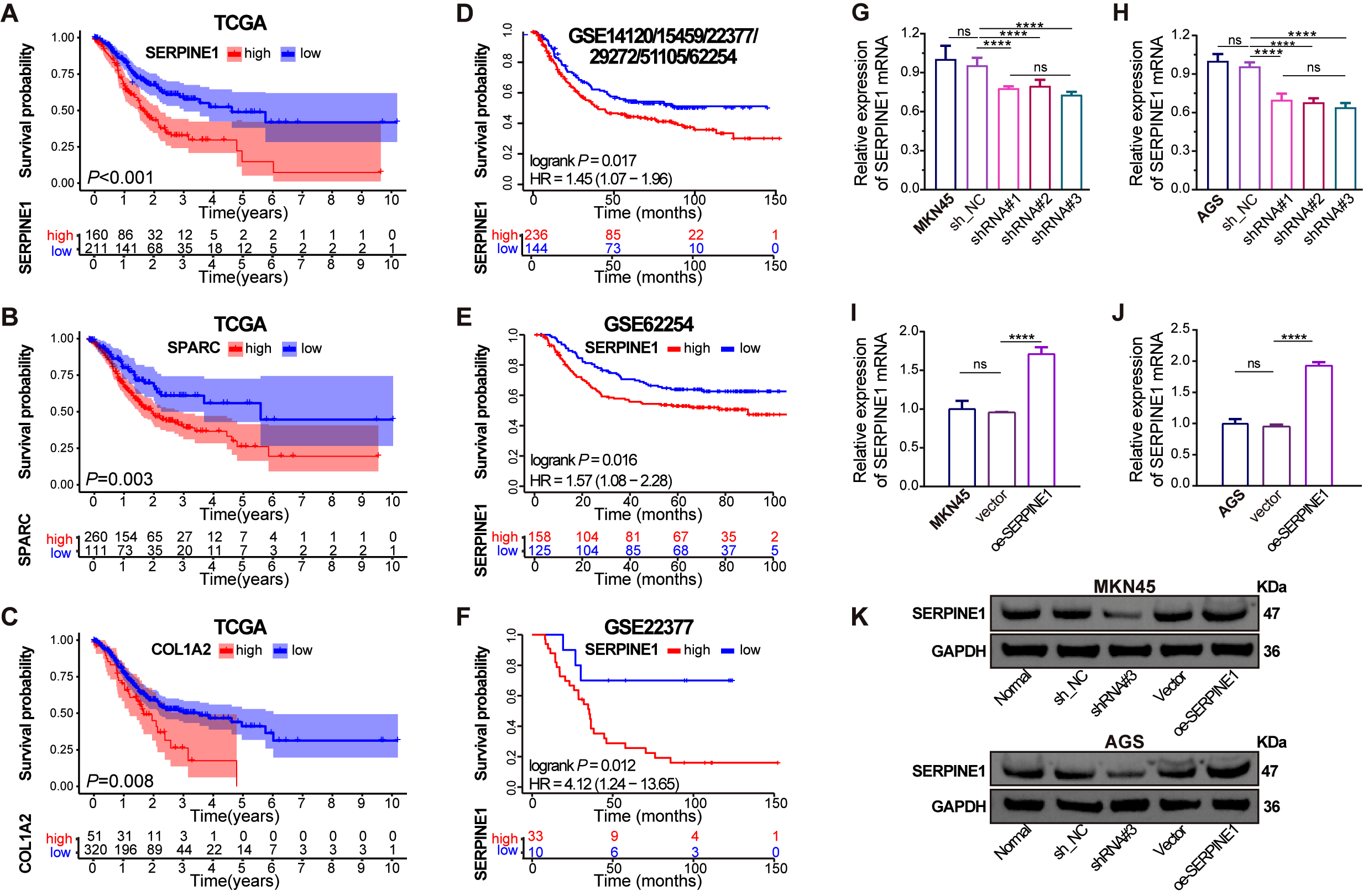
**

**Supplementary fig.1** (A-F) Kaplan-Meier survival curves of the prognosis-related module genes in TCGA cohort and GEO datasets. qRT-PCR (G-J) and western blotting (K) validated *SERPNE1* expression in GC cells transfected with shRNA lentiviral vectors for stably silencing *SERPNE1* and transient *SERPNE1* overexpression plasmids.

**
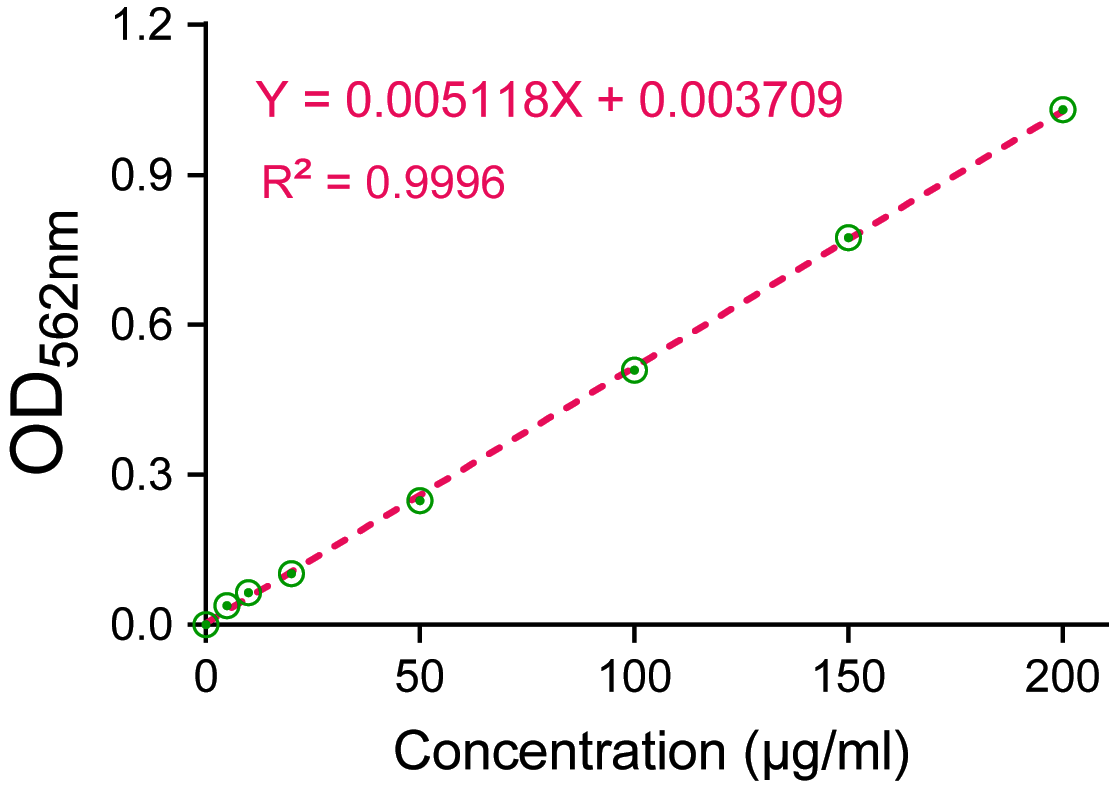
**

**Supplementary fig.2** Standard curves of the BCA assay.

**Supplementary table 1 Exosome protein concentration determined by BCA assay**

| **Cell** | **Group** | **average**  **OD value** | **absolute**  **OD value** | **1:10 dilution of**  **exosome (μg/mL)** | **exosome**  **concentration (μg/ml)** |
| --- | --- | --- | --- | --- | --- |
| **MKN45** | Normal | 0.1195 | 0.0379 | 6.71 | 67.12 |
|  | shNC | 0.1200 | 0.0384 | 6.81 | 68.10 |
|  | shSERPINE1#3 | 0.1205 | 0.0389 | 6.90 | 69.02 |
|  | vector | 0.1204 | 0.0388 | 6.88 | 68.76 |
|  | oeSERPINE1 | 0.1200 | 0.0384 | 6.80 | 67.97 |
| **AGS** | Normal | 0.1197 | 0.0381 | 6.75 | 67.52 |
|  | shNC | 0.1198 | 0.0382 | 6.76 | 67.58 |
|  | shSERPINE1#3 | 0.1204 | 0.0388 | 6.88 | 68.82 |
|  | vector | 0.1200 | 0.0384 | 6.80 | 68.04 |
|  | oeSERPINE1 | 0.1197 | 0.0381 | 6.75 | 67.45 |

**Primer sequences**

| Symbol |  | Primer sequence (5’-3’) |
| --- | --- | --- |
| SERPINE1 | F | ATCCTTGCCCTTGAGTGCTTGTTAG |
|  | R | AGTGGCTGGACTTCCTGAGATACG |
| let-7g-5p | F | GCGTGAGGTAGTAGTTTGTACAGTT |
|  | R | Universal reverse primer (Takara) |
| TGF-β | F | CCAGAGAGGTTAAGGGAGGAGTTC |
|  | R | AGTGCTAGGATTACAGGCGTGAG |
| IL-10 | F | CTTGCTGGAGGACTTTAAGGGTTAC |
|  | R | CTTGATGTCTGGGTCTTGGTTCTC |
| Arg-1 | F | CCCTTTGCTGACATCCCTAATGAC |
|  | R | TTCTTCTTGACTTCTGCCACCTTG |
| iNOS | F | CTCAGAGTACAGCAAGTGGAAGTTC |
|  | R | GAAAGCAGGAAGCCAGCAGAC |
| TNF-α | F | AAAGGACACCATGAGCACTGAAAG |
|  | R | AGGAGAAGAGGCTGAGGAACAAG |
| GAPDH | F | CACCCACTCCTCCACCTTTGAC |
|  | R | GTCCACCACCCTGTTGCTGTAG |
| U6 snRNA | F | GGAACGATACAGAGAAGATTAGC |
|  | R | TGGAACGCTTCACGAATTTGCG |
| let-7g-5p promoter site -1692~-1420 (chr3: c52270669-52270941) | F | CAAATGCTTGAACTATGATAG |
|  | R | TTATCTCGAGACATGCAGGCA |
| GAPDH for CHIP | F | TACTAGCGGTTTTACGGGCG |
|  | R | TCGAACAGGAGGAGCAGAGAGCGA |

**Antibodies for western blot**

| **Antibody** | **Type** | **Vendor** | **Catalog No.** | **Dilution** |
| --- | --- | --- | --- | --- |
| Calnexin | Rabbit mAb | abcam | [ab133615](https://www.abcam.cn/ab133615.html) | 1:1000 |
| CD63 | Rabbit mAb | abcam | ab134045 | 1:1000 |
| CD81 | Rabbit mAb | abcam | ab109201 | 1:1000 |
| GAPDH | Rabbit pAb | Immunoway | YM0294 | 1:2000 |
| JAK2 | Rabbit pAb | Immunoway | YT2426 | 1:1000 |
| PAI1 (SERPINE1) | Rabbit pAb | Immunoway | YT3569 | 1:1000 |
| phospho-JAK2 | Rabbit pAb | Solarbio | K009379P | 1:1000 |
| phospho-STAT3 | Rabbit pAb | Immunoway | YO0250 | 1:1000 |
| SOCS7 | Rabbit mAb | abcam | ab133677 | 1:1000 |
| STAT3 | Rabbit pAb | abcam | ab68153 | 1:1000 |
| TSG101 | Rabbit mAb | abcam | ab125011 | 1:1000 |
| Tubulin | Rabbit pAb | Immunoway | YT4780 | 1:2000 |
